# Seasonality in bipolar disorder, analysis of an inpatient unit

**DOI:** 10.1192/j.eurpsy.2025.1052

**Published:** 2025-08-26

**Authors:** M. I. Chaves, J. Garcês Marques, F. Varino, C. Siopa, A. I. Duarte

**Affiliations:** 1 Psychiatry, ULS Santa Maria, Lisbon, Portugal

## Abstract

**Introduction:**

Bipolar disorder is a chronic mental health condition characterized by alternating periods of mania, depression, and mixed states. The timing and recurrence of these mood episodes may be influenced by external factors, including seasonal variations. Previous research has highlighted potential seasonal patterns in the onset of manic episodes, with environmental factors such as changes in light exposure, temperature, and circadian rhythms playing a role. Comprehending these temporal patterns is paramount, as they provide critical insights into the underlying mechanisms of mood dysregulation and can inform the development of more targeted and effective clinical interventions for individuals with bipolar disorder.

**Objectives:**

The primary objective of this study is to determine whether manic and mixed episodes in bipolar disorder show a seasonal pattern. Specifically, this study aims to investigate whether specific times of the year are associated with a heightened incidence of hospitalizations for manic or mixed episodes.

**Methods:**

A retrospective analysis was conducted on patient records from an inpatient psychiatric unit over four years. Inclusion criteria were a primary diagnosis of bipolar disorder and admission due to a manic or mixed episode. Data were categorized by month and season of hospitalization, and statistical analyses were performed to assess for significant seasonal variations.

**Results:**

Our study revealed a significant increase in hospitalizations for manic episodes during the spring and summer months, with 58% of manic episodes occurring during this period, and a secondary peak in autumn. Mixed episodes demonstrated less pronounced but still observable seasonal variation. Statistical analysis confirmed the presence of seasonality, with manic episodes more likely to occur during periods of increased daylight, while mixed episodes appeared more distributed across the year.

**Conclusions:**

The findings indicate that manic episodes in bipolar disorder follow a distinct seasonal pattern, peaking in spring and summer. Although mixed episodes are less strongly correlated with seasonality, some seasonal trends were observed. These results highlight the significance of considering environmental and seasonal factors in the management of bipolar disorder. Further investigation into the underlying mechanisms of these patterns could improve preventative care and inform the development of personalized treatment strategies.

**Disclosure of Interest:**

None Declared

